# The double-edged sword role of fibroblasts in the interaction with cancer cells; an agent-based modeling approach

**DOI:** 10.1371/journal.pone.0232965

**Published:** 2020-05-08

**Authors:** Zarifeh Heidary, Jafar Ghaisari, Shiva Moein, Shaghayegh Haghjooy Javanmard

**Affiliations:** 1 Department of Electrical and Computer Engineering, Isfahan University of Technology, Isfahan, Iran; 2 Regenerative Medicine Research Center, Isfahan University of Medical Sciences, Isfahan, Iran; 3 Department of Physiology, Applied Physiology Research Center, Isfahan Cardiovascular Research Institute, Isfahan University of Medical Sciences, Isfahan, Iran; Universidade de Sao Paulo, BRAZIL

## Abstract

Fibroblasts as key components of tumor microenvironment show different features in the interaction with cancer cells. Although, Normal fibroblasts demonstrate anti-tumor effects, cancer associated fibroblasts are principal participant in tumor growth and invasion. The ambiguity of fibroblasts function can be regarded as two heads of its behavioral spectrum and can be subjected for mathematical modeling to identify their switching behavior. In this research, an agent-based model of mutual interactions between fibroblast and cancer cell was created. The proposed model is based on nonlinear differential equations which describes biochemical reactions of the main factors involved in fibroblasts and cancer cells communication. Also, most of the model parameters are estimated using hybrid unscented Kalman filter. The interactions between two cell types are illustrated by the dynamic modeling of TGFβ and LIF pathways as well as their crosstalk. Using analytical and computational approaches, reciprocal effects of cancer cells and fibroblasts are constructed and the role of signaling molecules in tumor progression or prevention are determined. Finally, the model is validated using a set of experimental data. The proposed dynamic modeling might be useful for designing more efficient therapies in cancer metastasis treatment and prevention.

## Introduction

The tumor microenvironment (TME) as the surrounding milieu of tumor cells is consisted of different types of components such as extracellular matrix (ECM), blood vessels, immune cells, adipose cells, and fibroblasts [1, 2]. Interactions among tumor stromal cells and cancer cells lead to maintenance and growth of the tumor tissue [3, 4]. Fibroblasts are one of the abundant factors in TME which have a major impact on tumor behavior [5, 6]. Fibroblasts have been overlooked despite their essential role in tumorigenesis in a context-dependent manner. Indeed they have been seen in two fundamental forms inside the TME, Normal Associated Fibroblasts (NAF) and Cancer Associated Fibroblasts (CAF) [7–9]. Although, the original sources of CAFs in different cancer types remain elusive, there is also some evidence that in specific type of cancers, CAFs exist in different subtypes based on their spatial niche within TME [10, 11]. Considering original and functional heterogeneity of CAFs, they are supposed to support growth and invasiveness of cancer cells, whereas NAFs inhibit these features [12, 13]. So, fibroblasts can be regarded as Janus-faced components of the TME and understanding the kinetics of their interactions with cancer cells is crucial for cancer treatment.

Over the last decade, systems biology has revolutionized our understanding from kinetics of complex biological behaviors by application of mathematical approaches. In this regard, agent-based modeling as a methodology that focuses on interactions among the elements of the system is an appropriate mathematical tool to study biological systems. Furthermore, this approach gives the opportunity to understand the behavioral kinetics of a tissue as a well-defined population [14–16]. Agent-based modeling has been used as a valuable tool in tumor computational biology [17]. Many aspects of tumor biology such as adaptation to microenvironment, the process of angiogenesis, the tumor cell ECM interaction, response to chemotherapy, the effects of hypoxia, and metastasis and invasion have been incorporated and investigated in agent-based models [15]. Gay et al have introduced agent-based modeling and its possible uses in the dynamics of innate immune response and systemic inflammatory response syndrome [18].

Agent-based modeling has been applied for determining the role of gene-protein interactions, cell phenotypes and molecular signatures [19–21]. Also, a number of mathematical models based on this method have been proposed for determining the role of cancer stem cells, platelet and tumor cell interactions in cancer metastasis [22, 23]. Furthermore, tumor immune response including immunotherapy has been modeled using hybrid and agent-based modeling approaches recently [24, 25]. Stochastic agent-based model using cellular automata formalism has been used for modeling immune-tumor interaction and suggest its significance to control tumor development [26]. In a recent study, we integrated bifurcation analysis with agent-based modeling to elucidate macrophage fate-determination and population patterns [27]. In the case of tumor and its milieu interactions there are a limited number of studies for example in [28] the interaction between a tumor and its surrounding stroma subpopulations investigated to recognize their role on the emergence of drug resistance and tumor growth by using a minimal ordinary differential equation (ODE) model based on exponential growth dynamics. Also, molecular rules that control the cancer cells and adjacent fibroblasts interactions were defined using a mathematical model based on singular value decomposition approach in [29]. Nevertheless, the mutual conversation among fibroblasts and cancer cells and the switching behavior of fibroblasts in cancer metastasis due to the intracellular regulatory signaling and intercellular communication have never been investigated from the agent-based modeling point of view.

In the current study, we present an agent-based model of the contradictory effect of fibroblasts on tumor suppression and progression as a switching behavior. At first, a modeling framework have been made to mathematically represent the dynamic biological system. In order to unravel the unknown parameters of the model a computational parameter estimation approach was applied based on model outputs best fitting with experimental data [30]. Finally, after simulation of the model with an appropriate software, validity of the outputs is evaluated by comparing them with a different measured dataset obtained from experiments.

To analyze how fibroblast switching behavior serves the tumor development, a mathematical model has been built. The model is composed of two agents; a fibroblast cell and a cancer cell. The agents have different dynamics modeled by nonlinear ODEs and they communicate with each other through intercellular signaling. The underlying mechanisms which are described by systems of differential equations are based on transforming growth factor β (TGFβ) and leukemia inhibitory factor (LIF) pathways as well as their crosstalk. These factors regulate growth, differentiation, migration and apoptosis in many cell types. They are also responsible for switching behavior in different stages of cancer development. Despite presence of other fibroblast activator molecules in the TME, considering TGFβ and LIF signaling pathways seems to be sufficient to model the interactions among fibroblasts and cancer cells due to their consecutive roles in the promotion of fibroblasts activation. Because of limited number of time points and noisy measurements, we benefited from advantages of Hybrid Unscented Kalman Filtering (HUKF) approach to estimate the unknown parameters. For this purpose, a gene expression profiling results of a microarray dataset (GSE6653) from Gene Expression Omnibus (GEO) database has been applied as observations [30–33]. Finally, the model represents outputs such that they are in accordance with experimental data. In our knowledge, this is the first study which uses agent-based modeling framework to describe switching behavior of the fibroblasts in the TME. In addition, this model can be used for solid tumors that have invasive characteristics due to the role of fibroblasts in the development of metastasis.

## Materials and methods

### Agent-based model for dual effect of fibroblast on cancer cell

Fibroblasts in NAF and CAF forms play different roles in the interaction with cancer cells. To describe the fibroblast switching behavior, a nonlinear dynamic model of the intracellular reactions in cancer cell and fibroblast as well as intercellular interactions between two cell types was built simultaneously. Activation of resident fibroblasts is induced by numerous cytokines released by cancer cells such as TGFβ and LIF [34–38]. TGFβ is one of the main factors secreted by cancer cell and fibroblast which involves in tumor growth, progression and metastasis [39]. When this factor releases in the TME, it binds to its receptor on the cell surface and activates intracellular signaling pathway through phosphorylation of SMAD2/3. Then, collaboration of phosphorylated SMAD2/3 and SMAD4 finally leads to expression of downstream genes [38]. Target genes of this pathway such as LIF and C-X-C motif chemokine 12 (CXCL12) play principle functions in cancer cell proliferation and migration [40, 41]. Furthermore, it is proposed that upon activation of TGFβ pathway, transcription factors such as SNAIL are activated which promote epithelial to mesenchymal transition (EMT) [31]. There is also an inhibitory SMAD (SMAD7) that inhibits SMAD2/3 phosphorylation which upregulates in TGFβ pathway and makes a negative feedback loop [31]. LIF cytokine also contributes in normal fibroblast reprogramming into CAF and reinforces invasive phenotype [34, 35]. It initiates Janus Kinase/ signal transducer and activator of transcription proteins (JAK/STAT) signaling pathway. It has been demonstrated that JAK/STAT pathway leads to pro-invasive phenotype in fibroblasts in the interaction with cancer cells [35]. Furthermore, crosstalk between TGFβ and LIF signaling pathway is a remarkable phenomenon which we have considered in the model. The effect of crosstalk between the two signaling cascade is both positive and negative. In other words, JAK/STAT in the LIF pathway upregulates target genes of SMAD2/3 in TGFβ pathway [42] including SMAD7 as well as CXCL12 which have different influences in the process of fibroblast behavior in the interaction with cancer cell. Although CAFs play a significant role in tumor growth, metastasis and invasion, NAFs prevent tumor progression by SLIT2 generation which inhibits some tumor promoting factors activity such as CXCL12 [43–45].

**Fig 1:**
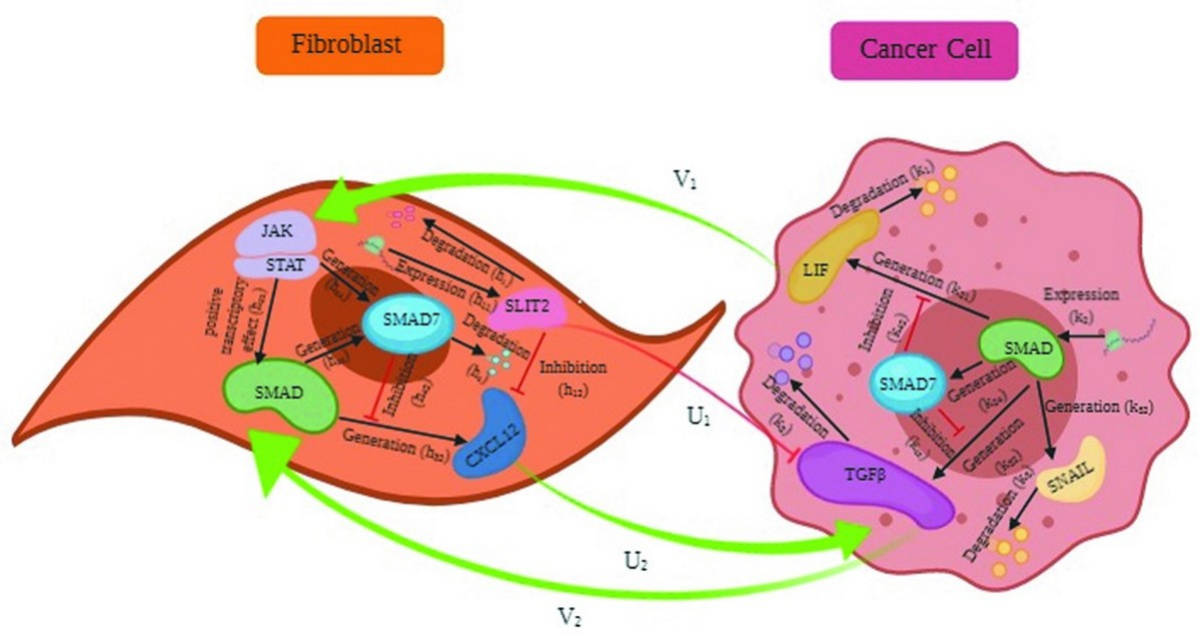
Molecular interactions between fibroblast and cancer cell. Mutual interactions between two cell types include basic reactions of TGFβ and LIF pathways as well as their crosstalk. Communications between two cell types are mediated through LIF, TGFβ, SLIT2 and CXCL12 as transmission signals **V**_**1**_, **V**_**2**_, **U**_**1**_ and **U**_**2**_ respectively.

As shown in Fig 1, the model consists of two agents corresponding to two different cell types. The first agent is cancer cell and the second is fibroblast. Dynamics of two agents are different but they interact with each other by their input and output signals. Although there are potentially hundreds of signaling molecules that are influencing these two cell types as well as the other cell types within the TME, we have considered two output signals for each agent which are the input signals of the other agent in the model. *V*_1_, *V*_2_ and *U*_1_, *U*_2_, are output signals of cancer cell and fibroblast which are the input signals of fibroblast and cancer cell, respectively.

The dynamic model is based on ordinary differential equations. Ordinary differential equations are used for quantitative modeling of biochemical reactions [46]. They represent how concentration of the reactants change with time. To analyze the reactions in which two or more reactants are involved in, the law of mass action is used. If the reaction is *A*+*B*→*C*, the law of mass action is as (1):

$$\frac{d[C]}{dt}=k[A][B]$$ (1)

Where [*A*], [*B*], and [*C*] are the concentrations of the A, B, and C and *k* is a constant which is called the reaction rate [47].

### Dynamic equations of the model

The cancer cell dynamic is a state space model of five differential equations which describe the important reactions in the TGFβ and LIF pathway as (2).

$$\begin{array}{l} R_{1,1}:\;{\dot{X}}_{1}=k_{21}X_{1}X_{2}-k_{1}X_{1}\\ R_{1,2}:\;{\dot{X}}_{2}=k_{2}X_{2}-k_{21}X_{1}X_{2}-k_{24}X_{2}X_{4}-k_{32}X_{2}X_{3}-k_{42}X_{4}-k_{52}X_{2}X_{5}\\ R_{1,3}:\;{\dot{X}}_{3}=U_{2}-U_{1}+k_{32}X_{2}X_{3}-k_{3}X_{3}\\ R_{1,4}:\;{\dot{X}}_{4}=k_{24}X_{2}X_{4}-k_{4}X_{4}\\ R_{1,5}:\;{\dot{X}}_{5}=k_{52}X_{2}X_{5}-k_{5}X_{5}\\ V_{1}=X_{1}\\ V_{2}=X_{3} \end{array}$$ (2)

The reactions in (2) are involved in activation of fibroblast, so cancer cell transmits TGFβ and LIF as signaling molecules to fibroblast. The states of cancer cell dynamic are shown in Table 1. LIF and TGFβ are depicted as output signals of cancer cell agent and as input signals for fibroblast agent in Fig 1 (*V*_1_ and *V*_2_ respectively).

**Table 1:** States of cancer cell agent.

States	Corresponding Molecule
X_1_	LIF
X_2_	SMAD
X_3_	TGFβ
X_4_	SMAD7
X_5_	SNAIL

Similarly, the dynamic of the fibroblast is a five equations state space model as shown in (3).

$$\begin{array}{l} R_{2,1}:\;{\dot{Z}}_{1}=h_{11}Z_{1}-h_{1}Z_{1}\\ R_{2,2}:\;{\dot{Z}}_{2}=h_{32}Z_{2}Z_{3}-h_{12}Z_{2}Z_{1}\\ R_{2,3}:\;{\dot{Z}}_{3}=-h_{32}Z_{2}Z_{3}-h_{34}Z_{3}Z_{4}-h_{42}Z_{4}+h_{35}Z_{3}Z_{5}+V_{2}\\ R_{2,4}:\;{\dot{Z}}_{4}=h_{54}Z_{4}Z_{5}+h_{34}Z_{3}Z_{4}-h_{4}Z_{4}\\ R_{2,5}:\;{\dot{Z}}_{5}=-h_{54}Z_{4}Z_{5}-h_{35}Z_{3}Z_{5}+V_{1}\\ U_{1}=Z_{1}\\ U_{2}=Z_{2} \end{array}$$ (3)

This model describes how CXCL12 and SLIT2 are expressed in fibroblast. The states of fibroblast dynamic are shown in Table 2. In our model, CXCL12 and SLIT2 are considered as sign of progression and prevention of cancer cell metastasis, respectively. So, they are regarded as signaling molecules which are transmitted from fibroblast to cancer cell. As depicted in Fig 1, CXCL12 and SLIT2 are the output signals of fibroblast agent and the input signals of cancer cell agent (*U*_1_ and *U*_2_ respectively).

**Table 2:** States of fibroblast agent.

States	Corresponding Molecule
Z_1_	SLIT2
Z_2_	CXCL12
Z_3_	SMAD
Z_4_	SMAD7
Z_5_	JAK/STAT

To produce a compendious view of TGFβ and LIF pathway as well as their crosstalk, some reactions were retrieved from the literature to construct (2) and (3) model and also depicted in Fig 1. In dynamic (2) LIF is up-regulated downstream the pathway with rate *k*_21_ in the interaction with SMAD as a transcription factor [41]. It is also degraded with rate *k*_1_ (*R*_1,1_). SMAD is generated with rate *k*_2_ and then is get used in LIF, SMAD7, TGFβ and SNAIL expression with rates *k*_21_, *k*_24_, *k*_32_ and *k*_52_ respectively, because as well as SMAD7 and LIF, TGFβ itself is a downstream gene of its pathway, too [48]. The reaction is also composed of an equation with rate *k*_42_ which shows the inhibitory effect of SMAD7 on the transcription activity of the SMAD (*R*_1,2_) [49, 50]. In another reaction, the generation and degradation of TGFβ with rates *k*_32_ and *k*_3_ is shown. It also contains two input signals from fibroblasts. The first signaling molecule is *U*_1_ or SLIT2 which inhibits CXCL12 generation downstream of TGFβ pathway [43, 44]. The other input signal is *U*_2_ or CXCL12 with positive influence on TGFβ function in fibroblast activation (*R*_1,3_) [40]. The next equation in cancer cell dynamic (*R*_1,4_) describes the SMAD7 expression and its degradation with rates *k*_24_ and *k*_4_ respectively. In the last equation (*R*_1,5_), SNAIL is produced downstream the TGFβ/SMAD with rate *k*_52_ and degraded with rate *k*_5_.

Fibroblast dynamic contains reactions which are occurred in fibroblast cell. In the first equation, expression and degradation of SLIT2 with rates *h*_11_ and *h*_1_ is shown (*R*_2,1_). In *R*_2,2_ CXCL12 is expressed with rate *h*_32_ downstream the pathway by SMAD as a transcription factor [40] and SLIT2 inhibits its activity with rate *h*_12_ [43]. In the third reaction (*R*_2,3_), SMAD transcription function is enhanced in the interaction with JAK/STAT [42, 51] (rate *h*_35_). Also, in this reaction *V*_2_ or TGFβ which is the input signal from cancer cell is considered as the initiator of pathway [31]. SMAD function in CXCL12 and SMAD7 expression is shown with rates *h*_32_ and *h*_34_. This reaction also contains the inhibitory effect of SMAD7 on SMAD activity which is described by rate *h*_42_. The next equation in dynamic (3) illustrates the equations in which SMAD7 is involved. SMAD7 is expressed downstream TGFβ and also LIF pathway which are shown by rates *h*_34_ and *h*_54_ respectively [51]. In this reaction (*R*_2,4_), degradation of SMAD7 is also shown (*h*_4_). In the last reaction (*R*_2,5_), JAK/STAT activity in the SMAD7 expression (*h*_54_) and enhancement of SMAD function (*h*_35_) are described [51]. It also contains *V*_1_ or LIF signaling molecule from cancer cell, because JAK/STAT role in this pathway is started with LIF [34].

Fibroblast can be CAF or NAF due to its dynamics and input signals from cancer cell. We supposed that *h*_11_, *h*_32_ and *h*_12_ which are the expression rates of SLIT2, CXCL12 and inhibitory effect of SLIT2 on CXCL12 respectively are dependent on the input signals of the fibroblast (LIF and TGFβ). The relation is described as (4):

$$h=m_{0}+m_{1}x_{1}+m_{2}x_{3}+m_{3}x_{1}x_{3}$$ (4)

in (4) *m*_0_ is a constant, *m*_1_, *m*_2_ and *m*_3_ are the impact factors of LIF, TGFβ and TGFβ and LIF crosstalk on *h* value, respectively. So, we have three relationships for *h*_11_, *h*_32_ and *h*_12_ with different values for *m*_1_, *m*_2_ and *m*_3_. In the next section, unknown parameter values are obtained using a parameter estimation approach.

### Model parameter estimation

In computational biology, the procedures for determining unknown parameters are drawn to the use of control theory and specifically Kalman filters recently [32]. These approaches were primarily developed to estimate unobserved states of a dynamical system based on minimization of estimation error covariance but, by appropriate expansion of dynamic system, they can be used for parameter estimation, too [52, 53]. In this study, hybrid unscented Kalman filter method has been applied for state and parameter estimation from a set of experimental data (GSE6653) which is based on a gene expression profiling to model SMAD regulatory modules in ovarian surface epithelium cells [33]. We selected gene expression data of four genes including SMAD7, CXCL12, SLIT2 and SMAD3 at four time points from this dataset.

Although, Kalman filtering approaches are basically designed for linear systems, there are a number of methods based on its principle which are applicable to nonlinear state estimation. Amongst them, Extended Kalman filter (EKF) and unscented Kalman filter (UKF) are more common for parameter estimation in biological contexts [54]. In order to nonlinear state estimation, the system has been linearized in EKF algorithms but, UKF algorithm directly runs on a nonlinear system. In this regard, UKF is a more accurate and robust method over EKF [55] and we use this method in this study. Whereas the dynamic system model is continuous in time and the experimental data is discrete-time so, the method is called hybrid UKF or HUKF.

Suppose that we seek to estimate unknown parameters of a nonlinear dynamic system in the form (5):

$$\dot{x}(t)=f(x(t),\theta )$$ (5)

Where *x*∈*R*^*n*^ is state vectors, *θ*∈*R*^*q*^ is unknown parameter vector. Since unknown parameters are constant, we can expand the dynamic model to (6) in which they are considered as additional states with zero rate of change [32]:

$$\left\{ \begin{array}{c} \dot{x}(t)=f(x(t),\theta )\\ \dot{\theta }=0 \end{array}\right.$$ (6)

Now, the expanded nonlinear dynamic system is ready to employ HUKF algorithm for state and parameter estimation simultaneously. HUKF relies on an unscented transformation which is transformed statistics of variables such that they can propagate through estimation steps [56]. Two main steps of estimation are prediction and correction. In the prediction step, estimation is done using model data and in the correction step the priory estimate is improved employing measurement information [32, 56]. We used ekf/ukf MATLAB toolbox and make necessary changes to the related functions in order to perform HUKF algorithm to determine unknown parameters of dynamics (2) and (3) [57]. The results are presented in Tables 3 and 4. Comparison between outputs and estimations and additional model parameters are gathered in supplementary files S1 Fig and S1 Table, respectively.

**Table 3:** Parameters of cancer cell dynamic.

Description of the reaction	Parameter	Value (*houre*^−1^)
**Generation of LIF downstream the TGFβ Pathway**	*k*_21_	0.1
**Degradation rate of LIF**	*k*_1_	0.0015
**Expression rate of SMAD**	*k*_2_	0.0487
**Generation of SMAD7 downstream the TGFβ Pathway**	*k*_24_	0.012
**Generation of TGFβ downstream its Pathway**	*k*_32_	0.502
**Degradation rate of TGFβ**	*k*_3_	0.122
**Inhibitory effect of SMAD7 on the transcription of genes by SMAD**	*k*_42_	0.0115
**Degradation rate of SMAD7**	*k*_4_	0.0000805 [58]
**Generation of SNAIL downstream the TGFβ Pathway**	*k*_52_	0.32
**Degradation rate of SNAIL**	*k*_5_	0.00016

**Table 4:** Parameters of fibroblast cell dynamics. Three of these parameters are calculated according to (4) which can be found in S1 Table.

Description of the reaction	Parameter	Value (*houre*^−1^)
**Expression of SLIT2**	*h*_11_	Ref to S1 Table
**Degradation of SLIT2**	*h*_1_	0.0514
**Inhibitory effect of SLIT2 on CXCL12 expression**	*h*_12_	Ref to S1 Table
**Generation of CXCL12 downstream the TGFβ Pathway**	*h*_32_	Ref to S1 Table
**Generation of SMAD7downstream the TGFβ Pathway**	*h*_34_	0.00008
**Generation of SMAD7 downstream the LIF Pathway**	*h*_54_	0.01 [59]
**Inhibitory effect of SMAD7 on the transcription of genes**	*h*_42_	0.00015
**Degradation of SMAD7**	*h*_4_	0.109
**Positive effect of JAK/STAT3 on the transcription of genes by SMAD**	*h*_35_	0.0013

### Model validation

To verify the results of our model, the microarray dataset GSE17708 was retrieved from GEO database (https://www.ncbi.nlm.nih.gov/geo/). This dataset encompasses samples of A549 lung adenocarcinoma cell line under TGF-beta treatment which was performed to investigate gene expression changes essential for metastasis progression [60]. The quality of samples was assessed by hierarchical clustering as well as Principle Component Analysis (PCA) using heatmap and ggplot2 packages of R software (version 3.5.2). Finally, the dataset was reanalyzed by GEO2R tool of GEO database to compare TGFβ treated samples in each-time point with untreated control group.

## Results

**Fig 2:**
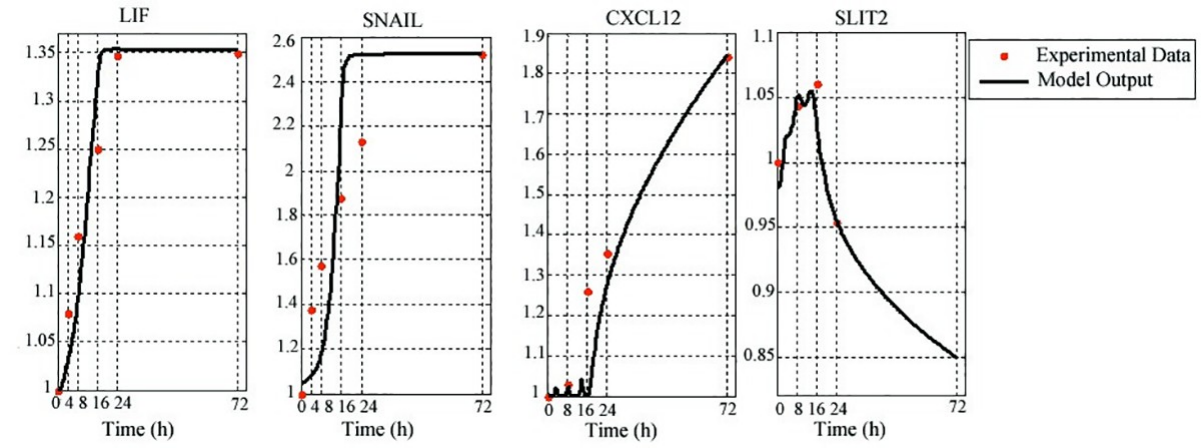
Model outputs of agent-based model compared with experimental data of GSE17708 dataset. The graphs show the concentration change of CXCL12, SLIT2, LIF and SNAIL over the time by continuous black line and experimental data in several time points by red dots.

Based on the above knowledge, we built an agent-based model of the interactions between fibroblast and cancer cell. Using MATLAB Simulink toolbox [61], the model is simulated. At the beginning of the simulation, the initial concentration of LIF and TGFβ are relatively low so fibroblast act as NAF which it means that SLIT2 concentration increases. SLIT2 increase is the sign of anti-cancer Characteristics of NAF in the tumor microenvironment. Then expression of LIF and TGFβ is increased by cancer cell and they are imported to the fibroblast as input signals *V*_1_ and *V*_2_. So, as shown in Fig 2 SLIT2 concentration decreases and CXCL12 concentration increases. Furthermore, expression of SNAIL as an EMT marker increased in cancer cell.

**Fig 3:**
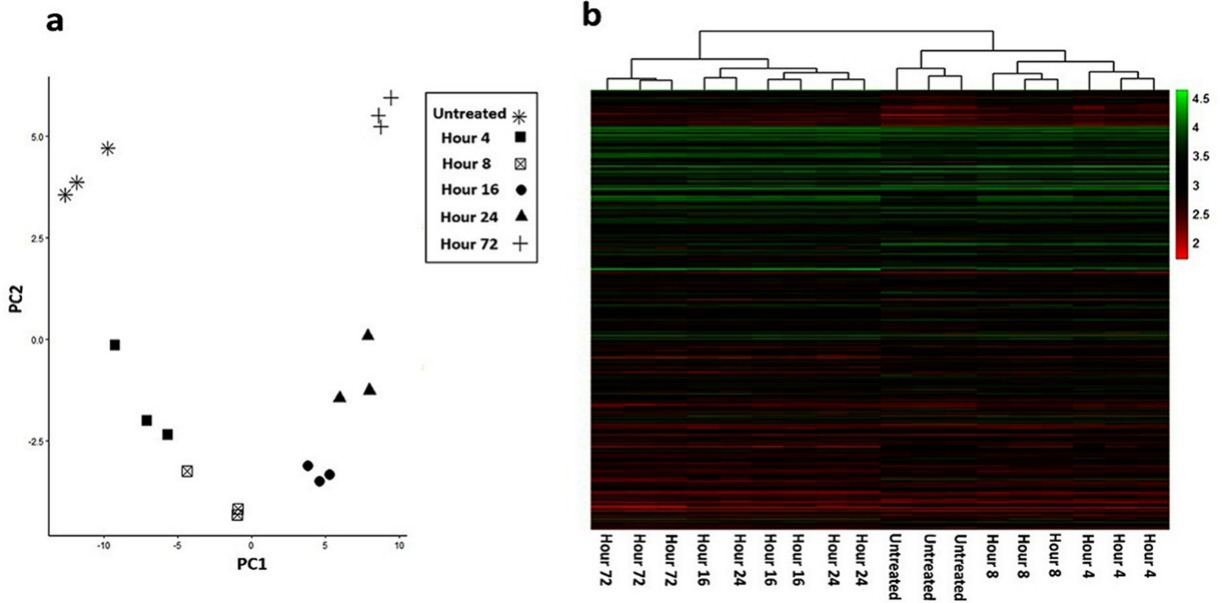
Principle Component Analysis (a) and heatmap clustering (b) demonstrated acceptable separation of samples in different time points.

Increasing *V*_1_ or LIF and *V*_2_ or TGFβ are signs of fibroblast activation and transformation of NAF to CAF, also overexpression of *U*_2_ or CXCL12 is sign of cancer promoting role of CAF and invasiveness of cancer cells. For verification of model outputs, microarray dataset GSE17708 was reanalyzed to determine the expression pattern of LIF, SLIT2, CXCL12 and SNAIL genes over the time. Hierarchical clustering and PCA demonstrated appropriate separation of samples in different time points (Fig 3), so these samples selected to compare with simulation results. The expression changes of selected genes indicate migratory and invasive phenotype of cancer cells.

As shown in Fig 2 Simulation results succeeded to reproduce the behavior of LIF, SLIT2, CXCL12 and SNAIL genes over the time with an acceptable proximity. We also computed normalized root mean square error (RMSE) for each of the above genes to estimate the difference among data values and simulation results samples. The RMSE is used to aggregate amount of errors for several times into a single measure of accuracy [62]. Normalized RMSE is the square root of the averaged squared error as shown in (7):

$$Normalized\;RMSE=\frac{1}{n}\sqrt{\sum_{i=0}^{n}{\left(x_{i}-\theta_{i}\right)}^{2}}$$ (7)

In (7), *x*_*i*_ is *i*th sample of simulation result, *θ*_*i*_ is *i*th sample of data and *n* is number of samples. Normalized RMSE values for CXCL12, LIF, SLIT2 and SNAIL genes are 0.05, 0.01, 0.01 and 0.1 respectively. These values indicate a relatively satisfactory validation for our model as well as, it could be also realized from Fig 2.

## Discussion

In the current study, we have generated a nonlinear mathematical model, in which internal interactions of cancer cell and fibroblast, as well as their intercellular communications are described. Modeling fibroblast and cancer cell interactions demonstrated fibroblast status change from NAF to CAF by cancer cell regulatory signals and its switching behavior in the TME. Fibroblasts switching behavior in the TME leads to dual effect on tumor progression. The model was constructed based on LIF and TGFβ pathways and also their crosstalk which are responsible for activation of normal fibroblasts and changing their status. Besides LIF and TGFβ, there are some key regulatory molecules such as CXCL12 and SLIT2 in the model which are cancer progression and prevention players, respectively. The model was successfully validated against experimental data.

SLIT is a family of secreted extracellular matrix proteins which act as tumor suppressors in normal condition. The SLITs play an important role in the cell migration, tissue development and establishment of the vascular network. Abnormalities or absences in the expression of SLITs has been reported in a variety of cancers [63, 64]. According to the model outputs, when a fibroblast is in normal status, SLIT2 increases and inhibits CXCL12 expression which leads to prevention of tumor growth and metastasis. The increased level of CXCL12, in the TME results in paracrine signaling via a feedback loop that promotes EMT and metastasis. It can also inhibit apoptosis through its upregulated receptors on tumor cells [65]. Fibroblasts in cancer associated status diminish SLIT2 production and subsequently by upregulation of CXCL12 as an agitation signal, promote metastatic behavior of tumor cells. So, the duplicity of fibroblast was considered as a two-faced spectrum and an agent-based model was built to describe the underlying mechanism in the proposed model.

In our proposed model, the dynamic behavior of cancer cell and fibroblast is described with nonlinear ordinary differential equations which are suitable tools to describe continuous biochemical interactions over the time. Furthermore, this modeling approach provides a holistic outlook into features and behavior of complex biological systems that leads to understanding control mechanisms governing them. Accordingly, generation of two different sets of nonlinear ODEs let us describe cancer cell and fibroblast dynamics and could determine the critical time-points of switching in SLIT2 and CXCL12 genes which are essential for fibroblast status change from normal to cancer associated type. Also, SNAIL as an EMT marker is considered in cancer cell agent and its expression downstream the TGFβ pathway shows the cancer cell invasion and metastasis.

Considering the fact that exact values of kinetic parameters for biochemical reactions are rarely available, whenever possible we used parameter values from previous studies. Other unknown parameters are estimated using HUKF algorithm. Furthermore, the model yielded results that are very close to experimental data, taking into account the key impact of agent-based structure and ODE modeling of significant reactions involved in fibroblast and cancer cell interaction. It should be mentioned that the biochemical reactions in our model are not limited to a particular context, so the model can be generalized for various types of cancer. For more accurate information about a specific type of cancer, general form of the proposed model can be used based on distinct tissue characteristics which may differ in some kinetic parameters.

Despite various experimental studies performed on cancer associated fibroblasts, the mechanisms behind their behavioral shift in relation to cancer cells remained undetermined. Our mathematical modeling could simulate the fibroblast and cancer cell communication and shed more light on fibroblast switching behavior in the interaction with cancer cells which has not been investigated quantitatively so far. Thus, from a mathematical point of view our suggested model is a pioneer in the study of this challenging biological phenomenon. Furthermore, consideration of different dynamics for agents is a notable strategy in agent-based modeling approaches that was applied in our study. In addition, our model describes the kinetic parameters of fibroblast signaling molecules (SLIT2 and CXCL12) with a polynomial which is dependent on cancer cell signaling factors (LIF and TGFβ); an attitude which is an innovative procedure in agent-based modeling structure. Accordingly, the model presented here can be regarded as an initial step to exploit mathematical approaches for deep understanding of how tumor microenvironment components influence cancer cells actions and therefore designing more effective treatment strategies.

## Supporting information

S1 Table Parameter values of *h*_11_, *h*_32_ and *h*_12_ which are the reaction rates of SLIT2, CXCL12 and inhibitory effect of SLIT2 on CXCL12 corresponding to *h* = *m*_0_+*m*_1_*x*_1_+*m*_2_*x*_3_+*m*_3_*x*_1_*x*_3_. In this relation *x*_1_ = LIF and *x*_3_ = TGFβ. The parameter values were determined using HUKF.
(PDF)

S1 Fig Hybrid unscented Kalman filter parameter estimation results based on four gene expression data. Black curves show real data and blue curves represent estimated values for corresponding genes including, SMAD, SMAD7, SLIT2 and CXCL12. Total error estimation for these four states is 0.06.
(TIF)
