# Supplementary material for: The double-edged sword role of fibroblasts in the interaction with cancer cells; an agent-based modeling approach
Source: PLoS One. 2020 May 8;15(5):e0232965. doi: 10.1371/journal.pone.0232965 (PMC7209353; doi:10.1371/journal.pone.0232965)
Supplement: S1 Table — In this relation x1 = LIF and x3 = TGFβ. The parameter values were determined using HUKF. (PDF) [file pone.0232965.s001.pdf]

parameter	m_0 $(houre^{-1})$	m_1 $(houre^{-1})$	m_2 $(houre^{-1})$	m_3 $(houre^{-1})$
h_{11}	0.0595	-0.215	-0.0113	-0.0016
h_{12}	0.00059	-0.099	-0.8502	-0.06202
h_{32}	0.0013	0.0012	5	0.0011
