# Supplementary figures and images for: The double-edged sword role of fibroblasts in the interaction with cancer cells; an agent-based modeling approach
Source: PLoS One. 2020 May 8;15(5):e0232965. doi: 10.1371/journal.pone.0232965 (PMC7209353; doi:10.1371/journal.pone.0232965)

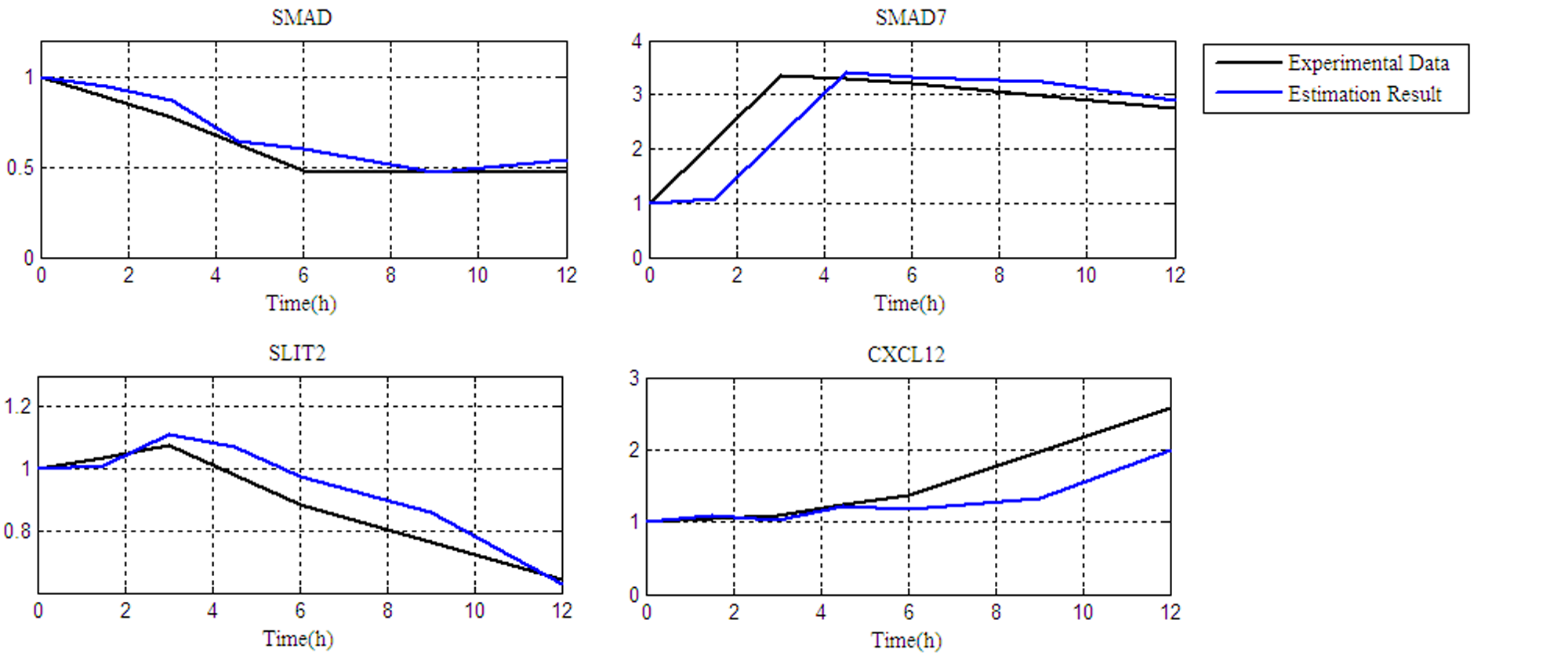

Supplement: S1 Fig — Black curves show real data and blue curves represent estimated values for corresponding genes including, SMAD, SMAD7, SLIT2 and CXCL12. Total error estimation for these four states is 0.06. (TIF) [file pone.0232965.s002.tif]
